# Increased Water Diffusion in the Parcellated Cortical Regions from the Patients with Amnestic Mild Cognitive Impairment and Alzheimer's Disease

**DOI:** 10.3389/fnagi.2016.00325

**Published:** 2017-01-11

**Authors:** Sung-Han Lin, Wen-Chuin Hsu, Shu-Hang Ng, Jur-Shan Cheng, Oleksandr Khegai, Chin-Chang Huang, Yao-Liang Chen, Yi-Chun Chen, Jiun-Jie Wang

**Affiliations:** ^1^Department of Medical Imaging and Radiological Sciences, Chang Gung UniversityTaoyuan, Taiwan; ^2^Graduate Institute of Clinical Medical Sciences, Chang Gung UniversityTaoyuan, Taiwan; ^3^Department of Neurology, Chang Gung Memorial HospitalTaoyuan, Taiwan; ^4^Dementia Center, Chang Gung Memorial HospitalTaoyuan, Taiwan; ^5^Department of Medical Imaging and Intervention, Chang Gung Memorial HospitalLinkou, Taiwan; ^6^Clinical Informatics and Medical Statistics Research Center, College of Medicine, Chang Gung UniversityTaoyuan, Taiwan; ^7^Department of Neurology, Chang Gung Memorial Hospital, Chang Gung University, College of MedicineTaoyuan, Taiwan; ^8^Department of Diagnostic Radiology, Chang Gung Memorial HospitalKeelung, Taiwan; ^9^Department of Medical Imaging and Radiological Sciences, Healthy Aging Research Center, College of Medicine, Chang Gung UniversityTaoyuan, Taiwan; ^10^Neuroscience Research Center, Chang Gung Memorial HospitalTaoyuan, Taiwan; ^11^Medical Imaging Research Center, Institute for Radiological Research, Chang Gung University/Chang Gung Memorial HospitalTaoyuan, Taiwan; ^12^Center for Advanced Molecular Imaging and Translation, Chang Gung Memorial HospitalLinkou, Taiwan

**Keywords:** Alzheimer disease, mild cognitive impairment, cerebral cortex, diffusion MRI, parcellation

## Abstract

**Background:** The loss of cortical neuron environment integrity is the hallmark of neurodegeneration diseases such as Alzheimer's disease (AD) and amnestic mild cognitive impairment (aMCI). To reveal the microenvironment changes in cerebral cortex, the current study aimed to examine the changes of mean diffusivity (MD) in parcellated brain among AD, aMCI patients and normal controls (NC).

**Methods**: Diffusion tensor imaging data with the whole brain coverage were acquired from 28 AD (aged 69.4 ± 8.2 year old), 41 aMCI patients (aged 68.2 ± 6.4 year old) and 40 NC subjects (aged 65.7 ± 6.4 year old). Subsequently, the MD values were parcellated according to the standard automatic anatomic labeling (AAL) template. Only the 90 regions located in the cerebral cortex were used in the final analysis. The mean values of MD from each brain region were extracted and compared among the participant groups. The integrity of the white matter tracts and gray matter atrophy was analyzed using the track-based spatial statistics and voxel-based morphometry approaches, respectively.

**Results**: Significant differences of MD were noticed both in aMCI and AD patients, in terms of the affected regions and the amount of increase. The hippocampus, parahippocampal gyrus and cingulum were the most significantly affected regions in AD patients. From all the 90 cerebral cortex regions, significant increase of MD in the AD patients was found in 40 regions, compared to only one (fusiform gyrus on the right) in aMCI patients. In the disease affected regions, the MD from aMCI patients is in state between NC and AD patients.

**Conclusions**: Increased MD in the specific regions of the brain shows the feasibility of MD as an indicator of the early stage cortical degeneration in aMCI and AD patients.

## Introduction

Alzheimer's disease (AD), a progressive neurodegenerative disease, is the most common form of dementia (Alzheimer's, 2014). In 2014, nearly 44 million population worldwide were affected by Alzheimer's or related dementia and expected to almost triple in 2050 (Prince et al., 2014). However, only 1-in-4 people with AD have been correctly diagnosed (Prince et al., 2014). Neuropathological studies showed that patients with AD are often accompanied with the deposition of amyloid and neurofibrillary tangle (NFT), which initiates from the temporal lobe and subsequently spreads out to the whole brain in the later stage (Braak and Braak, 1991). The accumulation of amyloid and NFT leads to neural death and brain shrinkage (Braak and Braak, 1991). Patients with AD suffer from the loss of brain functions, which typically starts with the impairment of the short-term memory in the early stage and extends to the long-term memory loss. The typical survival time from onset of AD to death varies from 4 to 8 years after the first diagnosis.

Mild cognitive impairment (MCI) refers to a specific group of patients who show deficits in the neuropsychological performance but do not satisfy the criteria of dementia (Petersen et al., 2001). Patients with amnestic type MCI (aMCI) have early memory complaints either with or without impairment in additional cognitive domains (Petersen et al., 1999). After a follow-up of 6 years approximately 80% of aMCI patients are diagnosed with AD (Petersen et al., 1999). It was noticed that this specific subgroup of patients will convert to AD at a rate 3~6 times higher when compared with the healthy subjects (Thompson et al., 2007). As a result, patients with aMCI are considered as a high-risk group of precursory AD (Prince et al., 2014). The understanding of the neuro-structural alterations in patients with aMCI, as related to the memory impairment, will potentially lead to a new insight of the underlying pathogenesis mechanism of AD.

Magnetic resonance imaging (MRI), a noninvasive imaging tool and free from ionizing radiation, has been used to study the extent of neuro-structural degeneration in patients with AD (Du et al., 2001; Thompson et al., 2007; Whitwell et al., 2008; Burton et al., 2009). The typical volumetric measurement in the brains of patients with AD reported atrophy in medial temporal lobe (MTL), hippocampus and the entorhinal cortex (Du et al., 2001; Burton et al., 2009). Similarly, atrophy in patients with aMCI also starts from hippocampus and entorhinal cortex (Whitwell et al., 2008). Post-mortem studies (Braak and Braak, 1991) showed that AD affected neurodegeneration sequence could initiate from medial temporal lobe and subsequently involve the whole brain. This similarity in the brain atrophy pattern further supports the hypothesis that aMCI could be a transition stage between the healthy status and AD (Ahmed et al., 2008). However, the brain atrophy by itself is not specific to the patients with AD. The morphological changes can only be detected at an advanced stage when the volume loss is no longer recoverable. Therefore, an image probe that is capable of detecting the subtle changes in the brain at an earlier stage of the disease is highly desirable.

Diffusion tensor imaging (DTI) allows for the measurement of micro-scale displacement of water molecules using MRI in a non-invasive manner. It could measure the water diffusivity in the brain *in vivo*, which is a reflection of the brain tissue integrity (Basser and Pierpaoli, 1996). Mean diffusivity (MD) is the average of water diffusivity measured along all directions (Basser and Pierpaoli, 1996). In a free and isotropic environment, the measured MD is approximately 3 × 10^−3^ mm^2^/s (Helenius et al., 2002). In the biological environment, water diffusion could be restricted and hindered. MD is approximately 0.89 × 10^−3^ mm^2^/s in the cortical gray matter and 0.70 × 10^−3^ mm^2^/s in white matter (Helenius et al., 2002).

Because the alteration in neuronal function might precede the structural atrophy, the current study proposed to investigate the value of changes in MD as an early sign of the neurodegeneration sequence in the natural course of Alzheimer Disease. Previous DTI studies on AD and/or aMCI mostly focused on either the changes in white matter tracts or the hypothesis-driven regions of interest such as hippocampus, entorhinal cortex and its neighborhood (Fellgiebel et al., 2004; Mielke et al., 2009). The most vulnerable white matter tracts involve uncinate fasciculus, superior longitudinal fasciculus, inferior longitudinal fasciculus and splenium of the corpus callosum (Liu et al., 2011; Wai et al., 2014), indicating a global influence of the brain from the aMCI and AD. Significantly increased MD in pathologically related structures can be found in hippocampus, cingulum and corpus callosum (Fellgiebel et al., 2004). However, the white matter degeneration could be secondary to the gray matter damage (Zhang et al., 2009). Therefore, the investigation into the general involvement of MD in the cortex is of great interest.

The MD value is a reflection of the balance between the different cellular contributions. Because the water diffusivity from the extracellular space could be larger than the intracellular component, the increased MD is often related to an increased extracellular volume (Duong et al., 1998), as a result of the collapse of neural integrity (Schaefer et al., 2000). The increased MD has been reported in the patients with neurodegenerative diseases, for example, Parkinson's disease, and was attributed to the microstructural alterations such as neural death (Lu et al., 2016). Because the neurodegeneration sequence often begin with the loss of neural integrity and result in the cell death in the end (Tripathi, 2012), the MD might showed increased sensitivity to the early sign of neurodegeneration.

In the present study, the hypothesis is that different extents of cortical damages between aMCI and AD can be detected by MD. Therefore, this study aimed to investigate the changes in MD as measured in cerebral cortex and assessed its potential as an early marker of neurodegeneration. Mean diffusivity from the parcellated cortical brain regions were measured. The differences between aMCI and AD in each region were examined. The correlation between the MD and cognitive performance was analyzed.

## Methods

The study was approved by the Institutional Review Board of Chang Gung Medical Foundation in Linkou, Taiwan, and complied with the Declaration of Helsinki. All participants gave written informed consent before participating the study.

### Participants

The participants were divided into three age-range matched groups, which consists of 40 healthy normal controls (NC, Male/Female = 22/18, aged 65.4 ± 6.2 year old), 41 patients with aMCI (Male/Female = 20/21, aged 68.3 ± 6.7 year old) and 28 patients with AD (Male/Female = 11/17, aged 68.5 ± 7.9 year old). All subjects were screened by a medical history review, physical examination, and neuropsychological testing. The physical examination, and neuropsychological testing was performed within 2 weeks of the MR examinations. Four key cognitive domains were assessed: memory, executive function, language and visuospatial skills. The neuropsychological assessments performed in this study including the Mini-Mental State Examination (MMSE), clinical dementia rating (CDR); word sequence learning-recall, visual reproduction, logic memory II in the memory function evaluation; semantic association of verbal fluency, Wisconsin Card Sorting Test (WCST) completed categories, Wechsler Adult Intelligence Scale-III (WAIS-III) digit symbol-scaled, WAIS-III digit span-scaled in executive function evaluation; 3-D block construction Models in visuospatial evaluation, and object naming test in the language function evaluation. Subjects with depressive symptoms were excluded by using either the Hamilton Depression Rating Scale (NC) or the Cornell scale for depression in dementia (AD and aMCI). The general exclusion criteria for MRI examination also applied. The Apolopoprotein E status were also examined if available.

The diagnosis of AD was made by using NINCDS-ADRDA criteria (McKhann et al., 1984). The MCI patients were determined by the criteria of Petersens et al. (Petersen, 2004). An aMCI patient was defined as patient with MCI and memory impairment. The healthy controls were identified as cognitively normal, judged by clinical assessment and neuropsychological testing. The demographic data are summarized in Table 1.

**Table 1:** **General characteristics of the study participants and neuropsychological evaluation scores**.

	**Control (*n* = 40)**	**aMCI (*n* = 41)**	**AD (*n* = 28)**
Mean age	65.4 (6.2)	68.3 (6.7)	68.3 (7.7)
Gender (Male/Female)	22/18	20/21	11/17
Education	10.4 (4.3)^*^	6.8 (5.1)	6.1 (5.2)^#^
Hypertension (w/o)	8/40	24/41	11/28
**APOLIPOPROTEIN E STATUS**			
ε4 carriers	0	9	4
ε4 noncarriers	22	14	12
Unknown	18	18	12
**MEAN GLOBAL SCORE**			
MMSE (Maximum 30)	28.6 (1.1)^*^	23.7 (4.9)^#^	14.6 (6.1)^#^^$^
Clinical dementia rating (CDR)	0.0 (0.0)^*^	0.5 (0.0)^#^	1.0 (0.6)^#^^$^
**MEAN MEMORY SCORE**			
Word sequence learning-recall	3.4 (1.7)^*^	0.2 (0.4)^#^	0.0 (0.0)^#^
Visual reproduction II	12.1 (2.7)^*^	8.0 (2.9)^#^	5.3 (1.3)^#^^$^
Logic memory II	12.3 (2.8)^*^	6.7 (3.9)^#^	2.4 (1.1)^#^^$^
**MEAN EXECUTIVE FUNCTION SCORE**			
Semantic association of verbal fluency	34.8 (7.1)^*^	28.7 (6.9)^#^	15.4 (7.4)^#^^$^
WCST-S completed categories	5.4 (1.1)^*^	3.4 (1.9)^#^	1.7 (2.9)^#^
WAIS-III digit symbol-scaled	10.7 (2.7)^*^	8.5 (3.3)	5.0 (2.7)^#^^$^
WAIS-III digit span-scaled	11.7 (2.8)^*^	9.8 (2.8)	7.6 (2.3)^#^
**MEAN VISUOSPATIAL SCORE**			
3-D block construction models	28.7 (0.6)^*^	26.9 (3.7)	19.1 (10.1)^#^^$^
**MEAN LANGUAGE SCORE**			
Object naming test	16.0 (0.0)^*^	15.9 (0.5)	13.1 (4.4)^#^^$^

*Mean (standard deviation); MMSE, Mini–mental state examination; WCST-S, Wisconsin Card Sorting Test-short form; WAIS-III, Wechsler Adult Intelligence Scale-III*;

* *indicates significant difference among three groups*.

# *indicates significant difference between patients and normal controls*.

$ *indicates significant different between aMCI and AD patients*.

### Image acquisition

Images were acquired using a 3T MR scanner (MAGNETOM Trio a TIM system, Siemens, Erlangen, Germany). T2 fluid-attenuated inversion recovery and T_1_-weighted magnetization-prepared rapid acquisition gradient echo (T1-MPRAGE) images were acquired to rule out the concomitant neurological disorders. T1-MPRAGE images were subsequently used in post-processing for data normalization. The following imaging parameters were used: repetition time (TR)/echo time (TE) = 2000 ms/2.63 ms, 160 axial slices of voxel size = 1 × 1 × 1 mm^3^, inversion time = 900 ms, flip angle = 9 degree. The single acquisition time was 4 min 08 s.

Diffusion weighted images were acquired using spin-echo echo planar imaging (EPI) sequence with the following parameters: TR/TE = 7324 ms/83 ms, 64 axial slices of voxel size = 2 × 2 × 2 mm^3^, *b*-value 1000 s/mm^2^ with the diffusion weighted gradient applied along 64 non-collinear directions. The single average acquisition time was 8 min 47 s.

### Image processing and analysis

#### The cortical parcellation

The procedure of image parcellation followed Lu et al. (2016) by homemade code in MATLAB (MATLAB 2013a. The MathWorks, Inc., Natick, Massachusetts, United States). The calculation and spatial normalization of the diffusion tensor were performed with Camino toolbox (Cook et al., 2006) (Microstructure Imaging Group—University College London) following the recommended procedure and parameters. The MD values were parcellated according to the standard automatic anatomic labeling (AAL) template following Ng et al. (2013). Briefly, non-diffusion weighted image (B0) from each individual was normalized to the ICBM 152 template from Montreal Neurological Institute (MNI) space 12-parameter affine transformation. The parameters used in the transformation were subsequently applied to MD of the same individual. The normalized MD was parcellated according to the AAL atlas. The mean values of MD from the 90 cortical regions were used in the final analysis.

#### Track-based spatial statistics

In order to determine the extent of white matter involvement, track-based spatial statistics (TBSS, part of FMRIB Software Library Smith et al., 2004) was performed following its recommended procedure and settings. The difference in white matter skeleton between groups was calculated using Randomized permutation program (with 100,000 times permutations) (Nichols and Holmes, 2002).

#### Voxel-based morphometry

Brain atrophy was assessed by Voxel-Based Morphometry (VBM) using the analysis toolbox from Statistical Parametric Mapping 8 (Wellcome Department of Imaging Neuroscience, London (Friston et al., 1994). The T1-MPRAGE images from all subjects were normalized to the standard MNI space and segmented into gray matter, white matter as well as cerebrospinal fluid within the same generative model. The normalized gray matter images were smoothed with a Gaussian kernel 8 × 8 × 8 mm FWHM.

#### Statistical analyses

All the statistical analyses were performed in PASW Statistics 21 (IBM Corp. Released 2012. IBM SPSS Statistics for Windows. Armonk, NY: IBM Corp.). The threshold of statistical significance was considered with Bonferroni *post hoc* correction. In the comparison of mean values among subject groups including the clinical neuropsychological scores and cortical MD values, one-way Analysis of variance (ANOVA) with *post hoc* multiple comparisons (Tukey honestly significant difference test). The statistical significance level was based on *p* < 0.05. Correction for multiple comparison was applied by using Bonferroni method (*p*-value < 0.0005, Bonferroni: 0.05/90). The correlation between cortical MD and the neuropsychological scores was performed with controlled aging effect after Bonferroni multiple comparison correction (*p*-value < 5.6E-5, Bonferroni: 0.05/900).

In TBSS, family wise error (FWE) correction was performed to correct for multiple comparison, with a threshold of *p* < 0.05. The abnormal white matter tracts were identified based on the atlas prepared at Johns Hopkins University (Wakana et al., 2004). In VBM, differences in gray matter between groups were assessed using two-sample *t*-test with threshold of *p* < 0.05 at false discovery rate (FDR).

## Results

### Clinical evaluation

Among the groups (NC, aMCI, and AD), no significant difference was noticed in gender, age and education level (Table 1). Compared to NC, AD patients had significant declination in all neuropsychological tests (*p* < 0.001). Compared to aMCI patients, AD patients showed reduced cognitive function in all tasks except for the learning-recall test of memory function, the completed categories test of executive function and the object naming test of language function. In addition, patients with aMCI had disturbed functions in the mean global and memory functions, semantic association of verbal fluency and the complete categories of Wisconsin card sorting test. No Apolipoprotein ε4 (ApoE4) carriers were found in NC. Nine of 41 aMCI patients and 4 of 28 AD patients were ApoE4 carrier.

### Gray matter changes in patients

**Figure 1:**
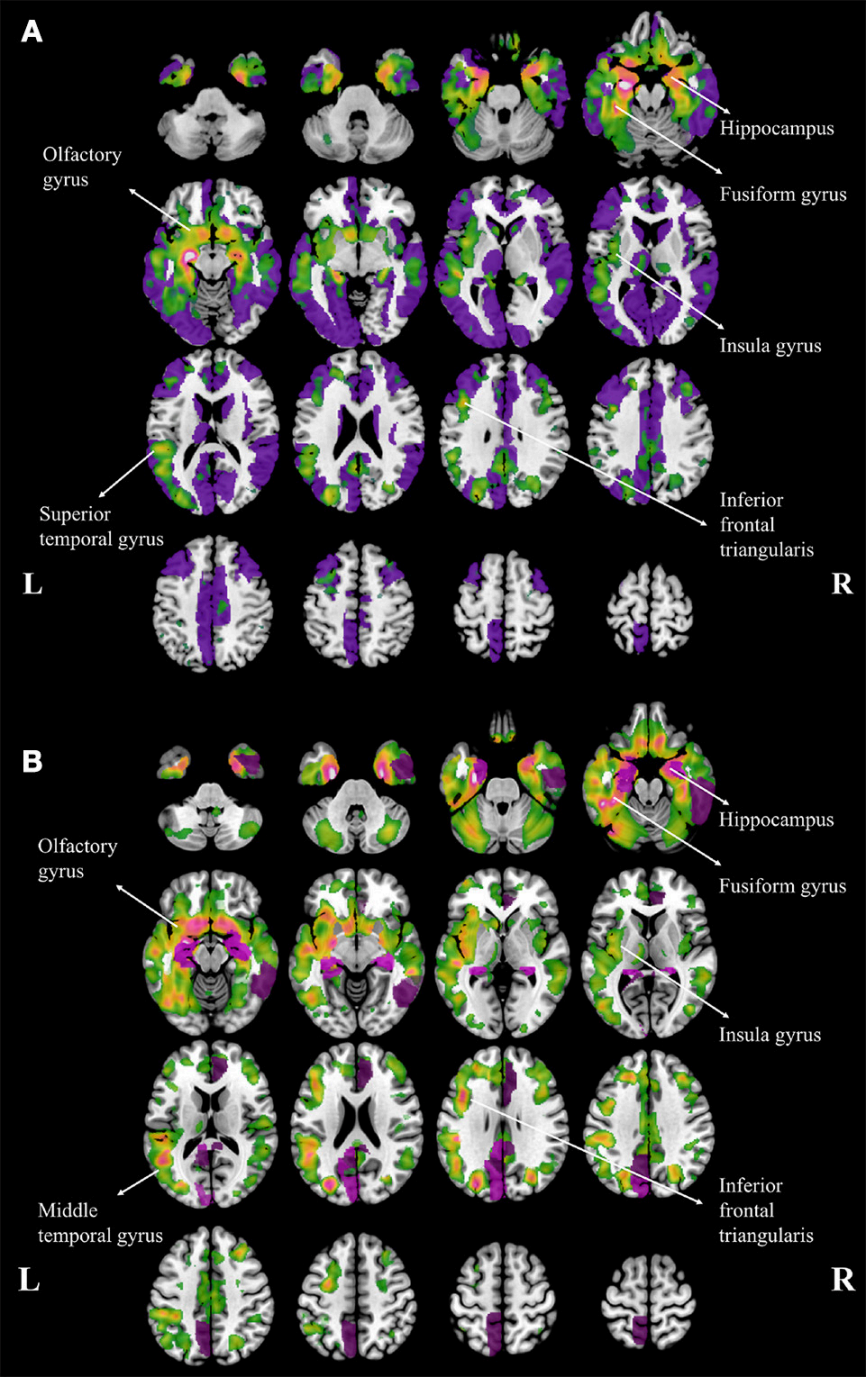
**Regions with increased mean diffusivity (MD) and gray matter loss in Alzheimer's disease (AD) patients, compared to normal controls (A) and amenstic mild cognitive impairment (aMCI) patients (B)**. The regions with significant increase MD are plotted with violet and the regions with gary matter atophy are plotted with warm colors, and the brighter color means the more severe atrophy in gray matter. The major atrophy regions were labeled with anatomy.

Compared to NC, aMCI showed increased cortical MD in the right fusiform. In contrast, wide spread regions (Figure 1A, violet colored, 39 AAL regions) with significant increase MD can be found in AD patients, mainly located within the temporal lobe and cingulate gyrus. When comparing AD patients to aMCI, significantly increased MD was noticed in 10 cortical regions (Figure 1B, violet colored), including the bilateral hippocampus, bilateral posterior cingulum, right anterior cingulum, right amygdala, right caudate and left cuneus.

Both patients with aMCI and AD performed volumetric loss in cortical regions. The atrophy regions in aMCI were found in bilateral hippocampus and right parahippocampal gyrus, when compared to NC. In contrast, atrophy regions were found in AD patients mostly in temporal, parietal and frontal lobes, including the hippocampus, insula, fusiform, superior temporal gyrus, olfactory, and the inferior frontal triangularis (Figure 1A, warm colors). In addition, AD patients showed atrophic regions mainly hippocampus, fusiform gyrus, insula, olfactory gyrus, middle temporal gyrus and inferior frontal triangularis when compared to aMCI (Figure 1B, warm colors).

### Increased cortical MD in AD and aMCI patients

**Figure 2:**
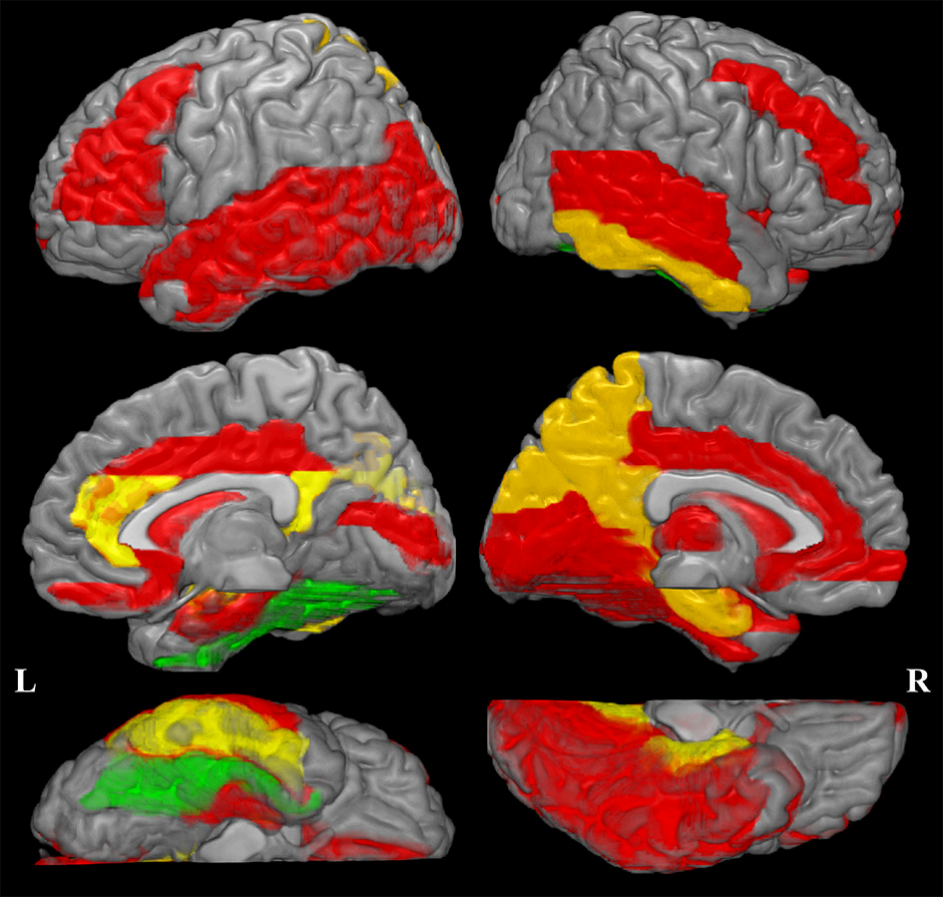
**Increased mean diffusivity (MD) pattern among patint with amenstic mild cognitive impairment (aMCI) and Alzheimer's disease (AD)**. Initial MD increase can be observed in aMCI patients only located in the right fusiform (violet color), when compared to normal controls (NC). Compared to aMCI patients, AD patients showed expended regions of increased MD (yellow color). In the later stage, increaed MD regions can be observed in every lobe of cortical brain in AD patinets (red color), compared to NC. This spatial distribution pattern of regions with increased MD is similar to the *post-mortem* pathology of amyloid deposition in AD patients (Braak and Braak, 1991).

**Figure 3:**
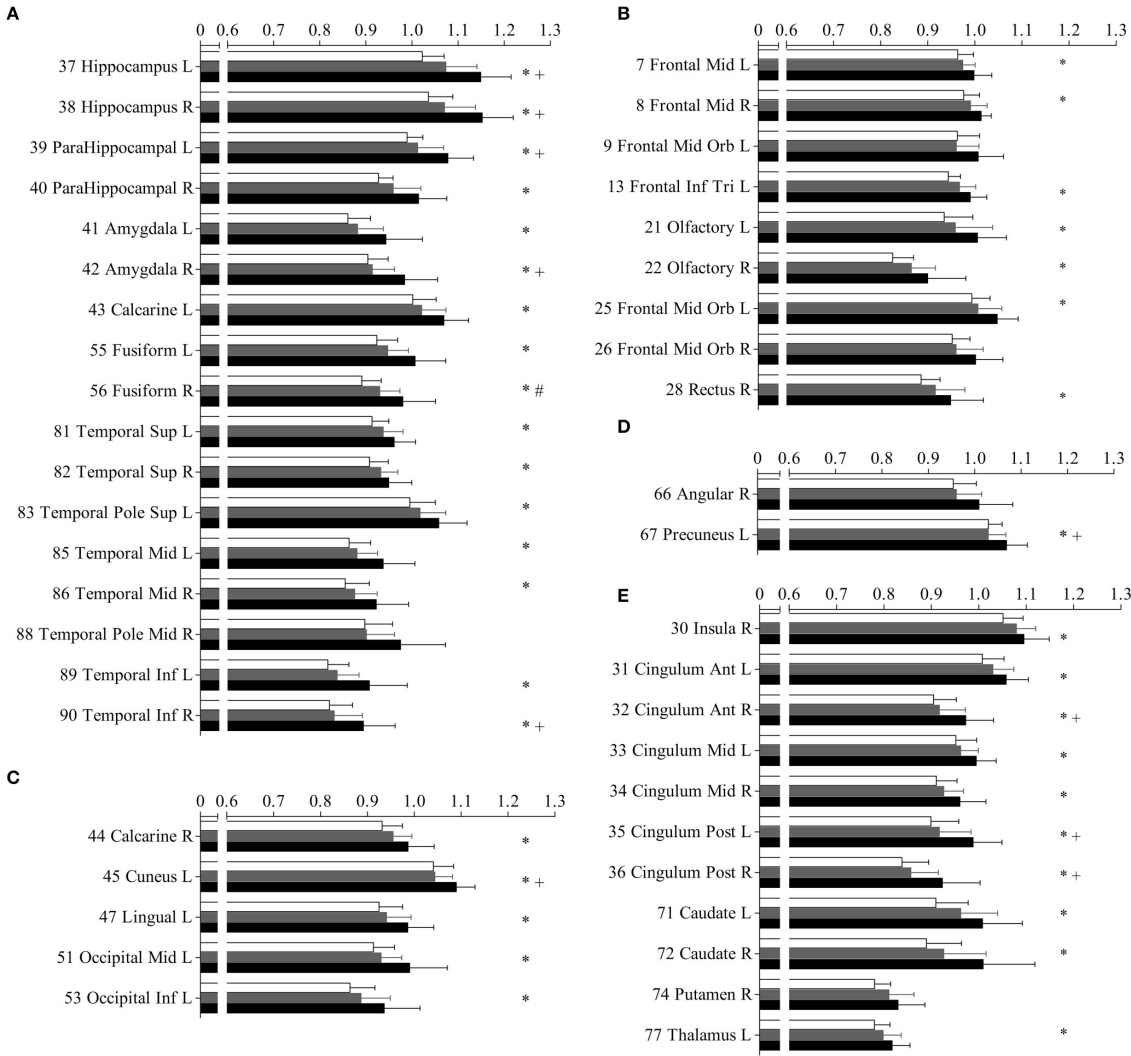
**Mean diffusivity (MD) in parcellated cortical regions**. The mean diffusivity in parcellated cortical regions with significant difference between Alzhimer's disease (AD) patients and normal controls (NC). Cortical MD of AD patients (solid), amnestic mild cognitive impairment (aMCI) (gray) patietents and NC (blank) are plotted. Stars indicate the significant difference between aMCI patients and NC (*p* < 0.0005). Pounds indicate the significant differences between aMCI and NC (*p* < 0.0005). Plus indicate the significant differences between aMCI and AD patients (*p* < 0.0005). The AAL cortical regions are arranged into temporal **(A)**, frontal **(B)**, occipital **(C)**, parietal **(D)**, and the deep nuclear **(E)**. The regions are numbered according to the anatomical automatic labeling template. Mean diffusivity is given in units of ^*^10^−3^ mm^2^/s.

The increased MD pattern among patients with aMCI and AD was showed in Figure 2. Initial MD increase can be observed in aMCI patients only located in the right fusiform (green color), when compared to NC. Compared to aMCI patients, AD patients showed expended regions of increased MD (yellow color), such as hippocampus, parahippocampus, amygdala, inferior temporal gyrus, cuneus, precuneus, anterior cingulum and posterior cingulum. In the later stage, increaed MD regions can be observed in every lobe of cortical brain in AD patinets (red color), compared to NC. Significant difference in the MD values were found in different cortical regions among the groups in comparison (Figure 3, stars: between AD and NC; pounds: aMCI and NC; plus: aMCI and AD). Although both patient groups have significantly increased MD in cortical regions, the amount of change and the affected regions from the aMCI patients is always less than AD. The left hippocampus was the site with the most significant increase (*p* < 0.00001, 12.51% increase in AD patients), when compared to NC. In the affected regions, MD increased by 7.52% in AD patients on average.

### White matter changes in patients

**Figure 4:**
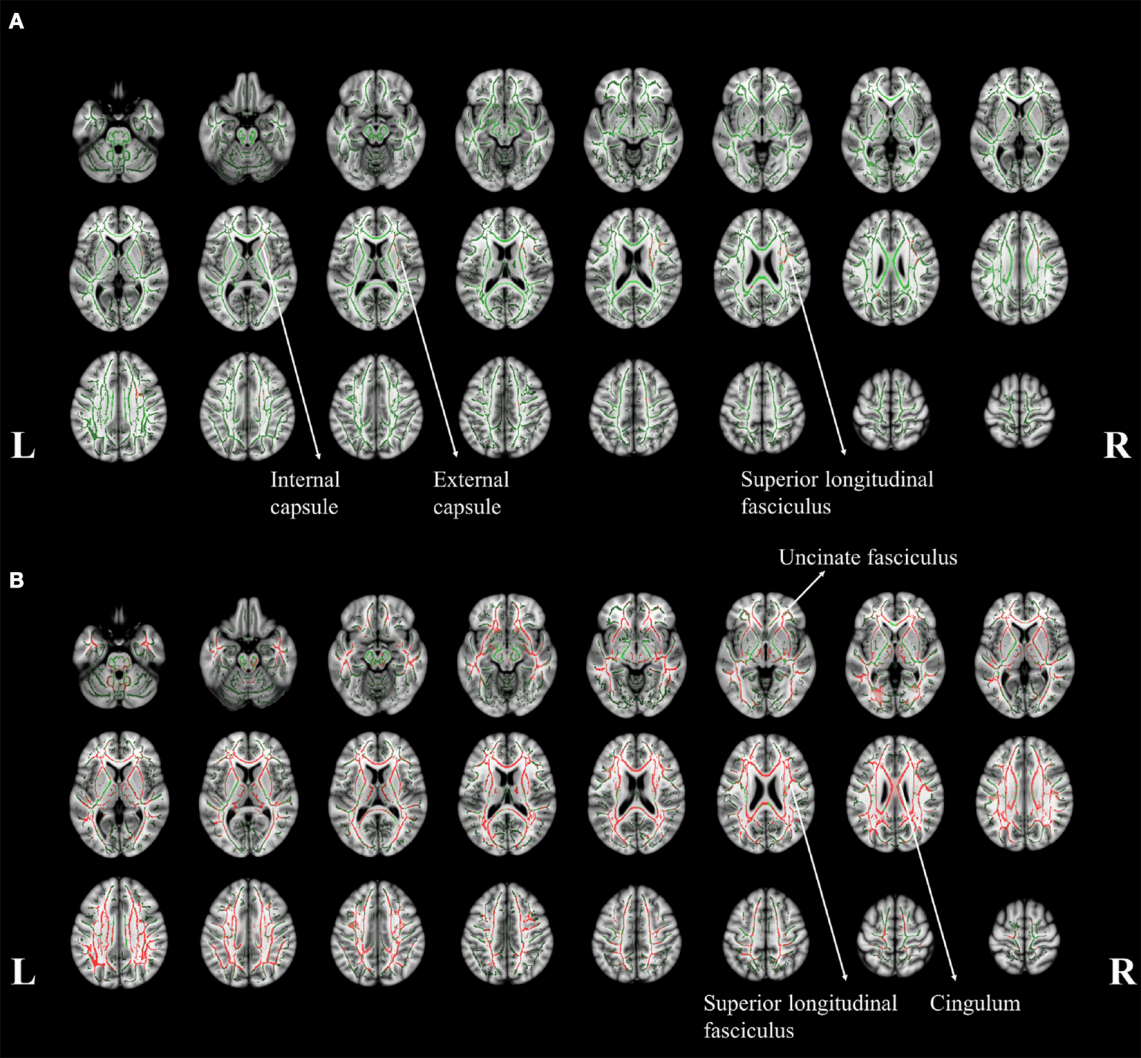
**The tract-based spatial statistics results of aMCI patients (A) and AD patients (B), when compared to normal controls**. The white matter tract skelotons are plotted in green lines and the pixels with significant MD increase are marked with red on tracts. The major regions with increased MD were labeled with anatomy.

To identify the difference in the involvement of white matter, the difference of TBSS in between aMCI and NC was shown in Figure 4. Significant changes can be noticed in right superior longitudinal fasciculus (SLF), internal and external capsule (Figure 4A). A general increase of MD was noticed throughout the whole brain (Figure 4B). However, no significant difference in white matter was found between the aMCI and AD patients.

### Correlation analysis in aMCI and AD patients

Table 2 summarizes the cortical regions with significant correlation between MD and neuropsychological scores. Hippocampus and its neighborhood in the temporal lobe, such as fusiform gyrus and inferior temporal gyrus, are the main regions with significant correlation the functions of interest.

**Table 2:** **Regions with significant correlation between MD and neuropsychological scores**.

**Neuropsychological scores**	**Cortical region (correlation coefficient, *p*-value)**	
MMSE	Olfa. R. (−0.41, 1.21E-5)	Cing. Ant. R. (-0.41, 9.62E-6)
	Cing. Post. L. (−0.46, 8.67E-7)	Cing. Post. R. (-0.45, 1.22E-6)
	Hipp. L. (−0.53, 6.90E-9)	Hipp. R. (−0.50, 4.83E-8)
	ParaHipp. L. (−0.54, 1.23E-9)	ParaHipp. R. (−0.44, 3.77E-6)
	Calc. L. (−0.43, 2.79E-6)	Calc. R. (−0.38, 4.79E-5)
	Ling L. (−0.43, 4.07E-6)	Occi. Mid. L. (−0.39, 4.13E-5)
	Fusi. L. (−0.58, 5.51E-11)	Fusi. R. (−0.53, 5.26E-9)
	Caud. R. (−0.42, 9.08E-6)	Thal. L. (−0.38, 5.09E-5)
	Temp. Inf. L. (−0.45, 1.51E-6)	
CDR	Olfa. R. (0.42, 1.03E-5)	Hipp. L. (0.44, 2.00E-6)
	Hipp.R. (0.54, 3.02E-9)	ParaHipp. L. (0.44, 2.25E-6)
	ParaHipp. R. (0.47, 6.07E-7)	Amyg. L. (0.41, 2.72E-5)
	Amyg. R. (0.49, 1.21E-7)	Fusi. L. (0.44, 1.54E-6)
	Fusi. R. (0.51, 3.59E-8)	Caud. L. (0.42, 7.39E-6)
	Caud. R. (0.43, 3.76E-6)	Temp. Inf. L. (0.40, 1.98E-5)
**MEMORY FUNCTION**		
Learning recall	Front. Inf. Oper. R. (−0.40, 4.06E-5)	Hipp. L. (−0.48, 5.82E-7)
	Fusi. R. (−0.40, 3.64E-5)	
Visual reprodiction	Hipp. L. (−0.46, 1.54E-6)	Hipp. R. (−0.41, 1.65E-5)
	ParaHipp. L. (−0.53, 1.00E-8)	ParaHipp. R. (−0.50, 7.91E-8)
	Amyg. L. (−0.40, 5.14E-5)	Fusi. L. (−0.52, 2.31E-8)
	Fusi. R. (−0.50, 9.87E-8)	Temp. Inf. L. (−0.40, 3.12E-5)
Logic memory	Hipp. L. (−0.56, 7.80E-10)	Hipp. R. (−0.48, 3.64E-7)
	ParaHipp. L. (−0.52, 1.49E-8)	ParaHipp. R. (−0.53, 1.11E-8)
	Amyg. L. (−0.54, 3.38E-6)	Calc. L. (−0.44, 2.91E-5)
	Fusi. L. (−0.50, 6.71E-8)	Fusi. R. (−0.50, 1.45E-7)
	Temp. Inf. L. (−0.45, 1.87E-6)	
**EXECUTIVE FUNCTION**		
Semantic association of verbal fluency	Front. Sup. Orb. L. (−0.43, 5.25E-6)	Front. Mid. L. (−0.40, 3.00E-5)
	Front. Mid. R. (−0.45, 1.63E-5)	Front. Mid. Orb. L. (−0.43, 6.80E-6)
	Front. Inf. Oper. R. (−0.41, 2.05E-5)	Front. Inf. Tri. L. (−0.41, 1.64E-5)
	Olfa. L. (−0.51, 4.25E-8)	Olfa. R. (−0.45, 2.71E-6)
	Cing. Ant. L. (−0.48, 2.93E-7)	Cing. Post L. (−0.47, 5.75E-7)
	Cing. Ant. R. (−0.45, 1.92E-6)	Hipp. L. (−0.50, 9.70E-8)
	Cing. Post R. (−0.43, 5.21E-6)	ParaHipp. L. (−0.49, 1.67E-7)
	Hipp. R. (−0.51, 3.21E-8)	Amyg. R. (−0.41, 2.53E-5)
	ParaHipp. R. (−0.59, 8.46E-11)	Calc. R. (−0.46, 9.59E-7)
	Calc. L. (−0.44, 3.83E-6)	Ling. L. (−0.48, 3.16E-7)
	Cuneus L. (−0.41, 1.61E-5)	Fusi. R. (−0.54, 7.71E-9)
	Fusi. L. (−0.62, 2.77E-12)	Caud. L. (−0.45, 1.97E-6)
	Precuneus L. (−0.44, 3.33E-6)	Temp. Sup. L. (−0.43, 6.01E-6)
	Caud. R. (−0.45, 1.80E-6)	Temp. Mid. L. (−0.57, 3.56E-10)
	Temp. Sup. R. (−0.41, 1.73E-5)	Temp. Pole Mid. L. (−0.42, 1.21E-5)
	Temp. Mid. R. (−0.47, 5.53E-7)	Temp. Inf. L. (−0.60, 2.42E-11)
	Temp. Pole Mid. R. (−0.41, 1.79E-5)	Temp. Inf. R. (−0.46, 1.16E-6)
WCST-S completed categories	ParaHipp. R. (−0.43, 1.11E-5)	
WAIS-III digit symbol-scaled	Hipp. L. (−0.45, 7.81E-6)	Hipp. R. (−0.44, 1.49E-5)
	ParaHipp. R. (−0.43, 2.64E-5)	Calc. L. (−0.42, 2.84E-5)
	Ling. L. (−0.41, 4.11E-5)	Fusi. L. (−0.49, 7.46E-7)
	Fusi. R. (−0.50, 6.06E-7)	
**VISUALSPATIAL FUNCTION**		
3-D block construction Models	Cing. Ant. R. (−0.39, 4.16E-5)	Cing. Mid. R. (−0.40, 3.05E-5)
	Cing. Post L. (−0.41, 1.61E-5)	Cing. Post R. (−0.45, 2.02E-6)
	Hipp. L. (−0.42, 1.25E-5)	Calc. L. (−0.47, 6.86E-7)
	Cuneus L. (−0.49, 1.39E-7)	Ling. L. (−0.41, 2.00E-5)
	Occi. Mid. L. (−0.55, 2.56E-9)	Occi. Inf. L. (−0.41, 1.94E-5)
	Fusi. L. (−0.54, 4.05E-9)	Fusi. R. (−0.48, 3.79E-7)
	Angular L. (−0.39, 5.07E-5)	Angular R. (−0.43, 5.53E-6)
	Precuneus L. (−0.43, 6.85E-6)	Temp. Mid. L. (−0.41, 1.71E-5)
	Temp. Inf. L. (−0.41, 1.66E-5)	Temp. Inf. R. (−0.41, 1.56E-5)
**LANGUAGE FUNCTION**		
Object naming test	Hipp. L. (−0.46, 9.50E-7)	Hipp. R. (−0.40, 2.91E-5)
	ParaHipp. L. (−0.51, 2.39E-8)	Amyg. L. (−0.41, 3.19E-5)
	Amyg. R. (−0.44, 4.28E-6)	Occi. Mid. L. (−0.49, 1.13E-7)
	Fusi. L. (−0.59, 5.47E-11)	Fusi. R. (−0.54, 6.17E-9)
	Parietal Sup. L. (−0.39, 3.46E-5)	Parietal Sup. R. (−0.39, 3.41E-5)
	Parietal Inf. L. (−0.47, 5.88E-7)	Angular L. (−0.41, 1.51E-5)
	Temp. Sup. L. (−0.39, 3.19E-5)	Temp. Pole Sup. L. (−0.47, 4.77E-7)
	Temp. Mid. L. (−0.60, 1.68E-11)	Temp. Mid. R. (−0.43, 5.46E-6)
	Temp. Pole Mid. R. (−0.46, 6.21E-7)	Temp. Inf. L. (−0.66, 2.74E-14)

*Regions with significant correlation between to MD and neurophyscological scores. R., right side; L., Left side; Front. Sup. Orb., superior frontal gyrus; Front. Mid., Middle frontal gyrus; Front. Inf. Oper, opercular part of inferior frontal gyrus; Front. Inf. Tri., triangular part of inferior frontal gyrus; Olfa., olfactory gyrus; Cing. Ant., anterior cingulum; Cing. Mid., middle part of cingulum; Cing. Post, posterior cingulum; Hipp., hippocampus; ParaHipp., parahippocampal gyrus; Amyg., amygdala; Calc., calcarine gyrus; Cuneus, cuneus gyrus; Ling., lingual gyrus; Occi. Mid., middle occipital gyrus; Occi. Inf., inferior occipital gyrus; Fusi., fusiform gyrus; Parietal Sup., Superior parietal gyrus; Parietal Inf., inferior parietal gyrus; Angular, angular gyrus; Caud., caudate nucleus; Thal., thalamus; Temp. Sup., superior temporal gyrus; Temp. Pole Sup., temporal pole of superior temporal gyrus; Temp. Mid., middle temporal gyrus; Temp. Pole Mid., temporal pole of middle temporal gyrus; Temp. Inf., inferior temporal gyrus*.

MD in the olfactory cortex and amygdala were significantly correlated with scores of Mini–Mental State Examination (MMSE) and Clinical Dementia Rating (CDR). The learning recall score of memory function was significantly correlated in regions located in the inferior frontal gyrus pars triangularis. The regions with correlation to semantic verbal fluency appeared to be scattered in the whole brain. The Wechsler Adult Intelligence Scale (WAIS-III) digit symbol-scaled score were significantly correlated with regions located in calcarine and lingual gyrus. In the visuospatial test, the 3-D block construction score significantly correlated with the cingulum, middle occipital, cuneus, lingual and angular gyrus. In the language function test, the object naming score was correlated with inferior parietal and angular gyrus. No significant correlation was found between education level and MD.

## Discussion

### Major findings

The current study reported an increased number of affected cortical regions and with growing difference, as the disease progressed from aMCI to AD. We measured the cortical mean diffusivity from two patient groups in comparison to the NC. Significant differences between AD and aMCI groups can be noticed in two aspects: the spatial pattern of the affected regions and the magnitude of increase. Although the atrophic pattern in AD suggested the whole brain involvement, the current study provided image based *in vivo* evidence that the microstructural damage might begin at an earlier stage. The spatial distribution suggested the involved regions might extend beyond the temporal lobe. The increase in mean diffusivity, a reflection of the severity in damage, indicated a widespread cortical impairment from MCI to AD. This spatial distribution pattern of regions with increased MD is similar to the *post mortem* pathology of amyloid deposition in AD patients (Braak and Braak, 1991). Furthermore, the affected region is consistent with that from the volumetric atrophy. Because the increase of MD is often attributed to an increase from the extracellular volume (Syková, 2004), as a result of the potential cell death, our observation might suggest a microstructural damage due to cellular degeneration or neural demyelination (Basser and Pierpaoli, 1996). Besides, significant correlations between the MD from the affected regions and the neuropsychological scores were observed.

### Increased MD in aMCI patients

In the aMCI patients, the current study observed significant atrophy located in the bilateral hippocampus, with increased MD in the peripheral region, noticeably fusiform gyrus. Only the right fusiform showed significant increase in MD (*p* = 0.0005, percentage increase of 4.35%). Similar increase of MD was noticed in bilateral hippocampi (Left side: *p* = 0.001, increase = 4.98%; Right side: *p* = 0.03, increase = 3.37%). Although the difference did not reach statistical significance, the percentage increase from left hippocampus is even higher than that in the right fusiform.

The white matter damage indicated that a more distant region could be affected such as SLF (Figure 4A). Post-mortem study showed that the brain degeneration in aMCI (Braak and Braak, 1991) started from the hippocampus and spread into the surrounding area in the later stages. The increased cortical MD in right fusiform might be related to the functions of word recognition and within-category identification (McCarthy et al., 1997). The finding is consistent with the reduced performance in verbal fluency and completed categories tasks in this specific group of patients. The gray matter atrophy pattern in the hippocampus might be related to the potential damage in memory formation and storage (Burgess et al., 2002), as observed in the impaired memory functions.

Increased MD of the right SLF might suggest declination in the microstructural integrity within this region. The SLF structurally connects with the temporal, occipital and frontal lobes, which is functionally associated with the memory formation and higher cognitive functions such as verbal fluency (Kamali et al., 2014). In addition, the loss of integrity of white matter tracts which connected to gray matter with memory function has been associated to AD-risk (Gold et al., 2012). Increased MD in SLF could be potentially more sensitive to neurodegeneration than the volumetric atrophy.

### Affected neural network in AD patients

Increased MD was observed in widespread cortical regions of the AD patients. The spatial distribution of the involved regions was similar to that in beta-amyloid deposition and neurofibrillary tangle (Braak and Braak, 1991). This similarity might suggest possible association between changes in MD and the disruption within the neuronal environment. This spatial pattern of increased MD is also consistent with gray matter atrophy, similar to the previous report (Burton et al., 2009). The region with the most significant increase of MD and the most severe volumetric atrophy are both located in the hippocampus. Because hippocampus and the entorhinal cortex are involved in consolidating information from short-term memory into long-term memory (Schwindt and Black, 2009), the dysfunction in these regions are the hallmark of the AD pathology (Braak and Braak, 1991; Du et al., 2001). The increase of MD in hippocampus might attribute to the memory loss in our study (Table 1).

The affected white matter tracts in AD mainly located in bilateral uncinate fasciculus, SLF, and cingulum (Figure 4B). These affected tracts involves in the connection between the frontal, temporal and occipital lobes in neocortex (Friederici, 2011). It can be noticed that the cortical MD in the surrounding area was significantly increased. This observation might suggest that a more extensive neural network might be involved, as reflected in the increase of MD. In addition, the degeneration of the cingulum involved the development of cognitive impairment in AD (Bozzali et al., 2012), which might refer to the significant cognitive dysfunction in our AD patient group.

### Enhanced cortical involvement from aMCI to AD

Compared with aMCI, more extensive cortical regions were involved in AD patients (one cortical region in aMCI patient and 40 in AD patients). Both the increase of MD and the involved regions suggest that the aMCI patients in the current study are in a transition state between the NC and AD patients. More severe brain degeneration in the more severe brain degeneration can be observed in AD patients when compared to aMCI. However, in term of white matter involvement, no significant difference between AD patients and aMCI can be found. This observation might support the fact that white matter changes could be secondary to the neurodegeneration in AD type dementia.

The current study noticed an overlap between the regions of gray matter atrophy and the more extensive and widespread areas of increased MD (Figure 1). The neurodegeneration process (Tripathi, 2012) begins with the loss of neural integrity, which subsequently evolve into massive cell death and shrinkage, and thus ultimately resulted in volumetric reduction and brain atrophy. The increase of MD is often attributed to the underlying microstructural damage. Because the spatial distribution pattern of the involved regions, as indicated by the increase of mean diffusivity, is similar to the *post-mortem* pathology of amyloid deposition in AD (Braak and Braak, 1991) and is more extensive than the atrophy map. It might suggest an increased sensitivity in the changes of MD than tissue atrophy. This observation certainly warrants new interest in further investigation in the future.

### Correlation between MD and neuropsychological performance

Hippocampus, fusiform and inferior temporal gyrus were mainly negative correlated with the cognitive evaluation scores. The previous studies reported that the hippocampus atrophy associated with not only loss of memory formation but also executive dysfunction such as non-memory cognitive impairment in early AD and aMCI patients (Burgess et al., 2002; Nagata et al., 2011). The current finding suggested that the degeneration of hippocampus and its surroundings were not only the landmark of aMCI and AD, but also involved the reduction of clinical cognitive performance in every aspect of evaluation including memory executive, visuospatial and language functions.

In addition to the hippocampus, different regions of cortical MD with significant correlation were noticed in different tasks for cognitive performance scores. Specifically, the memory function (the learning recall task) test showed negative correlation to MD in inferior frontal gyrus pars triangularis on left side, which has been known as a critical role in memory retrieval (Badre and Wagner, 2007). A scattered spatial pattern of cortical MD was also correlated with the semantic verbal fluency task of the executive function tests. The damage in cortical regions might interrupt the network of executive function including the ability of attention, planning and execution (Zhang et al., 2009; Nagata et al., 2011).

Deficits in visuospatial function in patients with aMCI and AD may suggest the loss of integrity of visual processing network. The middle occipital, cuneus and angular gyrus were noticed with significant correlation to the 3D block construction of the visuospatial test, which are critical roles involved the visuospatial information process (Bone, 2001). Interestingly, the regions with significant correlation between visuospatial function and cortical MD were mainly found on the left side. Although a lateralized brain network in visuospatial function were reported (Thiebaut de Schotten et al., 2011), the association between laterality of visuospatial function and cortical brain degeneration might need for further investigation.

### Effect of apolipoprotein E ε4 allele

Apolipoprotein ε4 (ApoE4) carriers could be found in patients with MCI and AD. The ApoE4 is the most widely known genetic risk factor for sporadic AD in different ethnic groups (Sadigh-Eteghad et al., 2012). However, Wai et al have reported a mild impact of the ApoE4 status on the extent of white matter disruption in the aMCI patients (Wai et al., 2014). The present study did not found significant trend between of the ApoE4 carrier and aMCI/AD. Number of ApoE4 carriers in aMCI/AD patient groups were smaller than non-carriers. Interestingly, the previous study has showed that the ApoE4 is less common in Chinese people than Caucasians or Japanese (Wong et al., 2005), which could explain the observation of leak of association between ApoE4 and AD/aMCI.

### Limitations

The major limitation in the current study is a compromised spatial resolution in a template-based parcellation approach, each of which consisted of multiple voxels. This might lead to the potential statistical type I error. In a template-based parcellation approach, the MD value is an average from all voxels within the region of interest. If the observed effect within the region is heterogeneous, the statistical significance might be affected. Furthermore, the inhomogeneous nature and limited number of aMCI participants might reduce the sensitivity of detection. To establish a relationship between MD and the amyloid deposition is an interesting and important issue, which will have to be investigated in the animal study in the future because biopsy is impossible in human neurodegeneration study. Secondly, the diagnosis remains clinical, based on psychometric evaluation (Petersen, 2004). In the study design, only age and gender in the participants were matched. This data inhomogeneity, as shown in MMSE, is one major limitation in the current study, which to an extent reflected the difficulty in the diagnosis of the neurodegenerative disease, specifically Alzheimer Disease. Objective evidences such as neuroimaging-based and genome-based might improve the accuracy of clinical judgment. Finally, because the APOE status remained uncertain for a significant amount of the participants because they did not provide the blood sample for examination, this might lead to concerns in the reliability of the result for the effects of APOE in neuroimaging.

## Conclusions

The current study identified a specific spatial pattern in the cortex of increased MD from patients with aMCI or AD. A correlation between MD and neuropsychological evaluations were reported between the functionally related cortical regions. The findings demonstrated the possibility of MD as an indicator at the early stage of cortical degeneration.

## Author contributions

SL, WH, CH, and JW contributed to the design of the study, acquired the data and drafted a significant portion of the manuscript. SN, YLC, and YCC supervised the data acquisition. SL, JC, and OK analyzed the data and drafted a significant portion of the manuscript and figures. JW and SL initiated the concept and the design of the study, acquired and analyzed the data and completed the manuscript.

## Funding

This work was supported by grants from the Ministry of Science and Technology Taiwan (MOST103-2325-B-182-001), Ministry of Education Taiwan (EMRPD1E1731) and the Chang-Gung Memorial Hospital (CMRPD1C0291-3 and CMRPD3D0011).

### Conflict of interest statement

The authors declare that the research was conducted in the absence of any commercial or financial relationships that could be construed as a potential conflict of interest.
